# Sexual experiences and emergency contraceptive use among female university students: a cross-sectional study at Wachamo University, Ethiopia

**DOI:** 10.1186/s13104-015-1070-7

**Published:** 2015-03-31

**Authors:** Tewodros Getachew Hailemariam, Tamene Tesfaye, Tedla Melese, Wondimu Alemayehu, Yeshialem Kenore, Yosef Lelamo, Tilahun Saul, Canaan Negash Seifu

**Affiliations:** School of Public Health, Wolaita Sodo University, Sodo, Ethiopia; School of Nursing & Midwifery, Wolaita Sodo University, Sodo, Ethiopia

**Keywords:** Sexually active, Emergency contraception, Female students, Ethiopia

## Abstract

**Background:**

Although unintended pregnancy rate is declining in both developed and developing countries, it remains higher in developing countries. Ethiopia is one country with a high prevalence of unintended pregnancy. In spite of this fact, very little is known about utilization of emergency contraception (EC) among young women. Therefore, this study aims to assess sexual experiences and emergency contraception use among female students at Wachamo University in Ethiopia.

**Methods:**

A cross-sectional study was conducted from March to April 2013 at Wachamo University in Ethiopia. A pretested self-administered questionnaire was used to assess sexual experiences and emergency contraception use among female students. The study participants (n = 424) were selected using a multistage sampling procedure. A simple random sampling technique was applied to select the study participants from a list obtained in registrar’s office. Data was entered into EpiInfo and exported to SPSS for analysis. Bivariate and multivariate logistic regression analyses were used to determine factors associated with emergency contraception use.

**Result:**

The majority of respondents (62.0%) were 20–24 years old and 31.4% were sexually active. Among sexually active, the mean (standard deviation) age at first sex was 18.22 (SD = 1.69). About one-half participants had high levels of knowledge about EC (49.8%) and positive attitudes towards EC (47.6%). Moreover, 44.4% of sexually active participants used EC at least once after unprotected sexual intercourse. The bivariate logistic regression revealed that age, marital status, religion, previous & current residence, parent’s educational status, knowledge about and attitude towards EC has a significant (P < 0.005) association with EC use. Furthermore, the multivariate analysis indicated that female students who have good knowledge, and ever got married were more likely to use EC than their counterparts (P < 0.05).

**Conclusions:**

Emergency Contraception use, knowledge about and attitude on Emergency Contraception were very low among female students. Overall, knowledge on EC and marital status were predictors for EC use. Thus, it is an indication that there is a need for health education and promotion programs in university set-up to improve EC use to prevent unintended pregnancies.

## Background

The unintended pregnancy rates are declining in both developed and developing countries, as indicated by nationally representative and small-scale surveys in 80 countries. However, of the 208 million pregnancies that occurred in 2008, 41% were unintended. Moreover, the rate of unintended pregnancy is higher in developing countries (57 per 1000 women between the age of 15–44) as compared to developed ones (42 per 1000 women between the age of 15–44) [1,2].

Unintended pregnancies can have a negative impact on women’s health in general, and on young female students in particular. A considerable number of women with unplanned pregnancies experience abortion and obstetric complications [3,4]. Globally, abortion is one of the significant causes of maternal mortality and accountable for 13% of all deaths related with pregnancy. About 45 million unintended pregnancies are terminated each year, and of these, 19 million are unsafely terminated. Moreover, 40% of unsafe abortions were carried out by young women aged 15 to 24 [5]. Though the unsafe abortion-related mortality ratio has declined worldwide, it continues to pose undue risks on women’s lives. The overall burden of unsafe abortion mortality also continues to be the highest in Africa [6].

Young un-married women are most vulnerable to this problem. Several studies have revealed that a significant proportion (21% to 79%) of young women are sexually active [7-9]. One recent study also found that unintended pregnancy is associated with age at the first sexual intercourse, use of protection during first sex, and awareness of the impact of abortion on future pregnancies [10].

Emergency contraception (EC) is one option for preventing unplanned pregnancy when it is available and properly used. However, it is largely underutilized worldwide [11-14]. In many low-income countries, women resort to unsafe abortion due to lack of knowledge about and access to EC. This also has a significant contribution to maternal morbidity and mortality [9,15].

According to Ethiopia’s Demographic & Health Surveillance (EDHS) data in 2011, the national prevalence of unintended pregnancy was found to be 24% [16]. Such unintended pregnancy could lead women to depression and other health outcomes [17]. On the contrary, very limited information is available about EC especially among female students at newly established universities of Ethiopia. Therefore, the aim of this study was to assess EC use and associated factors among female students at Wachamo University, one of the newly established universities.

## Methods

### Study design

We employed a cross-sectional study design to assess factors associated with emergency contraception use among female students at Wachamo University during the 2005 E.C (2013/2014) academic year.

### Study setting

The study was conducted at Wachamo University from March 23 until April 10, 2013. The University is situated at Hosanna, which is the administrative town of Hadiya Zone. It is found 235 km south of Addis Ababa, the capital of Ethiopia. The University was founded at its current location in 2012. According to the registrar’s office, it began its operation with 228 females and 310 male first batch students in December 05, 2012. While we are conducting the study, the university was teaching 1937 (1305 males and 632 females) first and second year students in 6 faculties and 25 departments.

### Study participants & sample size calculation

The study participants were first and second year female students at Wachamo University. The sample size was calculated using single population proportion formula, assuming a 95% level of confidence interval, 0.05 margin of error, and proportion of emergency contraception use of 28.7% [18]. After the correction of the primary sample size, we used a design effect of 2 to calculate the final sample size. Finally, after adding a 10% of non-response to the final sample size, the target sample size was 462 female students.

### Sampling procedure

A multistage sampling procedure was used to select study participants. First, students were divided in to two groups: first year and second year students. We then further divided these groups by department. From each department, participants were selected by simple random sampling, based on proportional allocation of the number of female students in each department.

### Measurement tool

We adopted an instrument (questionnaire) from previous studies on emergency contraception [9,18]. The questionnaire consists of five thematic areas: 1) socio-demographic information; 2) reproductive history; 3) knowledge of EC; 4) attitude towards EC; and 5) use of EC.

The knowledge section of the tool consists of seven items with response options of “No” = 0, “Yes” = 1, and “I don’t know” = 2. To compute knowledge score, we recoded the responses of knowledge variable into 0 and 1. Out of seven points, respondents who score the mean and above were labeled having “good knowledge” where as the rest were labeled as “do not have good knowledge” about EC.

On the other hand, respondents attitude towards EC was measured using six items rated on four point scales; 1) Strongly disagree, 2) disagree, 3) agree and 4) strongly agree. After reversing items, which are negatively stated statements, we computed the attitude score. The score was then recoded into another categorical variable using the mean score. Accordingly, for attitude, out of 24 points, those students who score the mean (15.57) and above were labeled as having “positive attitude” and the rest were labeled as having a “negative attitude” towards EC.

We checked the internal consistency of knowledge and attitude items in the tool using reliability test. The Cronbach’s alpha for seven items for knowledge and six items for attitude variables were 0.746 and 0.711 respectively.

### Data collection & analysis

A structured self-administered questionnaire was used to collect the data. Trained nurses supervised the data collection process. Data was entered using Epi Info(TM) 3.5.2, and then exported to SPSS 16.00 for analysis.

Descriptive statistics (frequency, percent, and mean with standard deviation) were drawn from data. We conducted a bivariate analysis to examine the potential associated factors of EC use. Then, all variables that were significantly associated (P < 0.1) with EC use in our bivariate analysis were considered in our multiple logistic regression models. Finally, variables that remained significant at (p < 0.05) in the multiple logistic regression model were identified as main predictors of EC use.

The overall findings were compiled and presented in tables and graphs with brief description. Odd ratios with 95% CI were reported to two decimal places and p-values less than 0.05 indicated statistical significance.

### Ethical issues

Ethical clearance was obtained from the College of Health Sciences & Medicine, Wolaita Sodo University. A formal request was submitted to Wachamo University and permission to conduct the study was obtained from the university. Individual consent was sought from study participants before they provided responses. All study participants were informed that participation was on a voluntary basis. Moreover, an anonymous questionnaire was used to maintain confidentiality.

## Results

Out of 462 respondents, a complete response rate of 91.77% was obtained. Thirty-eight students submitted questionnaires that were missing more than 30% of the questions. Therefore, we excluded the incomplete responses from analysis. Table 1 shows the socio-demographic characteristics of the respondents. The majority of respondents were single (85.1%), 20–24 years old (62.0%), and residing on campus (92.9%). According to the response of participants the mean (standard deviation) monthly pocket money of female student was 313.54 (SD = 227) Birr^a^.

**Table 1:** **Socio-demographic characteristics of regular female students, Wachamo University, Ethiopia**

**Characteristics**	**Number**	**Percent (%)**
**Age**		
15 – 19	129	30.4
20 – 24	263	62.0
25 – 29	32	7.5
**Marital status**		
Single/Never married	361	85.1
Married	47	11.1
Divorced	9	2.1
Separated	7	1.7
**Religion**		
Protestant	183	43.2
Orthodox	106	25.0
Catholic	52	12.3
Muslim	63	14.9
Others	20	4.7
**Place of residence before joining university**		
Rural	195	46.0
Urban	229	54.0
**Current place of residence**		
In the campus	394	92.9
Outside the campus	30	7.1
**Year of study**		
Year I	306	72.2
Year II	118	27.8
**Faculty**		
Health Sciences and Medicine	34	8.0
Natural Sciences	138	32.5
Social Sciences & Humanity	33	7.8
Engineering & Technology	143	33.7
Agriculture	35	8.3
Business and Economic	41	9.7
**Mother’s educational status**		
Illiterate (unable to read and write)	102	24.1
Primary (grade 1–8)	196	46.2
Secondary (grade 9–12)	75	17.7
College and Above	51	12.0
**Father’s education**		
Illiterate (unable to read and write)	61	14.4
Primary (grade 1–8)	101	23.8
Secondary (grade 9–12)	129	30.4
College and above	133	31.4

### Sexual experiences

Regarding sexual activity, 133 (31.4%) of study participants were sexually active prior to the study. Among sexual active participants, the mean (standard deviation) age at first sex was 18.22 (SD = 1.69), and 97 (72.9%) of them reported age at first sex prior to 19 years.

### Level of knowledge and attitude regarding EC

The study also examined knowledge and attitude of female students on EC. Results showed that 221 (49.8%) of our study participants did not show “good” knowledge about EC. Similarly, only 202(47.6%) of respondents showed a positive attitude towards EC.

### Emergency contraceptive use

**Figure 1:**
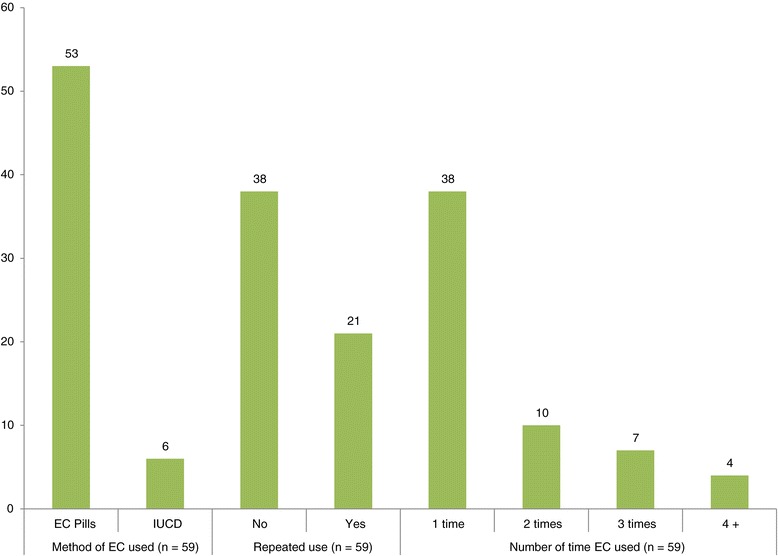
**Methods and repeated use of EC among regular female students Wachamo University, Ethiopia.**

**Figure 2:**
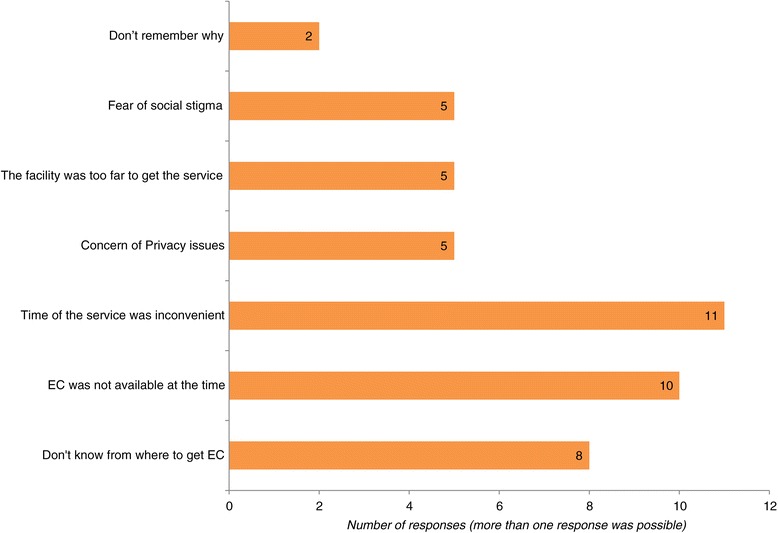
**Reasons for unable to use EC among sexually active female students after unprotected sex in Wachamo University, Ethiopia.**

Fifty-nine (44.4%) of sexually active female students reported using EC at least once after unprotected sexual intercourse. Of these, more than 35% of respondents used EC twice or more as depicted in Figure 1. Thirty-eight (28.6%) sexually active female students reported that they were unable to use EC when they were in need of it. The main reasons for not using EC after having unprotected sex are depicted on Figure 2.

### Factors associated with EC use

Based on bivariate analyses, age, marital status, religion, residence before joining university (rural or urban), current residence (on or off of the campus), mother’s educational status, father’s educational status, knowledge and attitude towards emergency contraceptive had a statistically significant (p < 0.05) association with EC use. After multivariate logistic regression analysis (Table 2) only marital status, religion, and knowledge of EC remain significantly associated with EC use.

**Table 2:** **Factors influencing use of EC among regular female students, Wachamo University, Ethiopia**

**Predictors**	**Crude OR (95% CI)**	**Adjusted OR (95% CI)**
**Age**		
≤20 Years old	1	1
>20 Years old	3.54 (1.95, 6.39)***	1.19 (0.54, 2.61)
**Marital status**		
Single/Never married	1	1
Ever Married	6.98 (3.77, 12.93)***	6.99 (3.09, 15.85)***
**Religion**		
Protestant	1	1
Orthodox	1.99 (0.98, 4.07)	0.85 (0.35, 2.05)
Catholic	3.26 (1.46, 7.26)**	1.81 (0.69, 4.73)
Muslim	1.03 (0.39, 2.73)	0.93 (0.29, 2.95)
Others (Adventist & Apostolic)	3.26 (1.05, 10.06)*	12.42 (2.68, 57.42)**
**Residence before joining university**		
Rural	1	1
Urban	2.15 (1.19, 3.87)*	1.67 (0.79, 3.50)
**Current place of residence**		
In the campus	1	1
Outside the campus	2.95 (1.28, 6.79)*	0.87 (0.27, 2.82)
**Class year**		
Year I	1	1
Year II	1.39 (0.78, 2.51)	1.09 (0.53, 2.29)
**Study background**		
Health & Natural Sciences	2.02 (0.84, 4.89)	2.427 (0.82, 7.16)
Social, Business & Economics	1	1
**Mother’s educational status**		
Illiterate (unable to read and write)	1	1
Primary (grade 1–8)	4.43 (1.52, 12.94)**	3.31 (0.83, 13.15)
Secondary (grade 9–12)	8.31 (2.69, 25.66)***	3.74 (0.81, 17.36)
College and Above	3.27 (0.88, 12.15)	1.61 (0.28, 9.27)
**Father’s educational status**		
Illiterate (unable to read and write)	1	1
Primary (grade 1–8)	4.36 (0.95, 20.02)	2.18 (0.37, 12.78)
Secondary (grade 9–12)	3.88 (0.86, 17.55)	1.22 (0.19, 7.42)
College and above	8.23 (1.89, 35.71)*	2.69 (0.42, 17.27)
**Pocket money/month (ETB)**	1.001 (1.000, 1002)	1.00 (0.99, 1.00)
**Level of knowledge**		
Not good	1	1
Good	9.41 (4.16, 21.28)***	8.51 (3.28, 22.12)***
**Level of attitude**		
Negative	1	1
Positive	3.17 (1.74, 5.77)***	1.78 (0.87, 3.64)

*Significant at 0.05, **Significant at 0.01, ***Significant at 0.000.

## Discussion

In our study, 31.4% of study participants reported having sexual intercourse at least once. This finding is comparable to other studies conducted on sexual behavior among students at Addis Ababa, Adama and Gonder universities [9,18,19].

Almost three-quarters of the study participants reported age at first sex before 19 years. This is the time, by which, young women need sex education to make informed decision. A number of studies have revealed that sex education is effective when teenagers and young adults are exposed to it before they become sexually active. It has shown to delay age at sexual initiation, reduce risk behaviors, and lower rates of adolescent pregnancy [20-23].

Successful use of EC requires accurate knowledge of and favorable attitude towards EC methods. Half of our study participants did not show either proper knowledge and/or positive attitude. The level of knowledge is a bit lower among our study participants. It is comparable with a study done in Uganda in which only 45.1% of female students heard about EC [24]. However, our finding on knowledge and attitude was low as compared to the results of Addis Ababa & Adama universities’ studies on EC [9,18]. This could be due to the fact that Wachamo University was newly established at the time of the study and only first and second year students were available to participate. Studies have shown that student’s knowledge on such matter improves with age and years of study at their campus [9,18,19]. Moreover, the difference could be due to measurement tools used to assess knowledge and attitude.

Emergency contraception use after unprotected sexual intercourse was found to be 44.4%. This is somewhat lower than rates of EC use reported at Adama University [18], and much lower than reported rates of EC use at Addis Ababa University (75%) and Bahir Dar University (73.4%) [9,25]. Furthermore, underuse of EC among unmarried sexually active women was reported by various Demographic and Health Survey reports from 2005–2009: 21.7%, 15%, 11%, and 10%, in Albania, Ukraine, Kenya, and Colombia, respectively [26-28]. This discrepancy could be due to availability of service within or near to these universities.

Among those who had used EC, more than one-third of respondents reported they had used EC twice or more. As evidence shows, women who are repeatedly using EC are more likely to start using regular contraception for the first time [29]. Thus, it is an opportunity for promoting regular contraception methods for those who have regular sexual relationships. A considerable number of sexually active respondents reported that they were unable to use EC due to a number of reasons, including 1) absence of EC at point of service; 2) inconvenient service time; 3) lack of knowledge on where to get the contraception; 4) distance to the service, 5) fear of stigma; and 6) concern for privacy.

A large number of factors were associated with EC use in our bivariate analysis. Knowledge and marital status were the strongest predictors of EC use that remained significant during a multivariate analysis. Respondents who had “good” knowledge about EC were almost 9 times more likely (OR 8.51, 95%CI: 3.28-22.12) to use EC than their counter parts, controlling for all other factors in the model. Moreover, those who were married were almost seven (OR 6.99, 95% CI: 3.09 - 15.85) times more likely to use EC as compared to those who were never married. Similar findings have been reported about female student populations elsewhere, implying that married students have better knowledge, when compared with unmarried students [19]. Moreover, lack of knowledge is one of the most important reasons among many reasons for underuse of EC [30,31].

## Conclusions

As University’s female students become more sexually active, they may engage in unprotected sexual intercourse. This could lead them to unintended pregnancy. This study revealed that EC use was very low among female Wachamo University students. Knowledge and marital status were major predicators of using EC among this target population.

### Endnote

^a^At the time of study, the conversion rate for Ethiopian Birr to USD($) was 1$ = 18.4894 Birr.
